# Sleep quality status, anxiety, and depression status of nurses in infectious disease department

**DOI:** 10.3389/fpsyg.2022.947948

**Published:** 2022-10-14

**Authors:** Shuangmei Xi, Yanmei Gu, Huimin Guo, Boxun Jin, Fengjuan Guo, Wenjing Miao, Lili Zhang

**Affiliations:** ^1^Department of Critical Care Medicine, Beijing Youan Hospital, Capital Medical University, Beijing, China; ^2^Department of Nursing, Beijing Youan Hospital, Capital Medical University, Beijing, China; ^3^Transplant Center, Beijing Youan Hospital, Capital Medical University, Beijing, China; ^4^Department of Emergency, Beijing Youan Hospital, Capital Medical University, Beijing, China

**Keywords:** infectious diseases, nurses, sleep quality, depression, anxiety

## Abstract

**Objective:**

To investigate the current status of sleep quality and influencing factors of clinical nurses in infectious disease hospitals, and to provide basis and reference for improving their sleep status and providing psychological support.

**Methods:**

Using convenience sampling method, clinical nurses from a tertiary hospital for infectious diseases were selected as the survey subjects in September 2021. General information questionnaire, Pittsburgh Sleep Quality Questionnaire (PSQI), Generalized Anxiety Disorder Scale (GAD-7), Depression Screening Scale (PHQ-9) were used for questionnaire surveys, and multiple linear regression was used to analyze the impact of decreased sleep quality in clinical nurses factor.

**Results:**

A total of 460 questionnaires were returned, of which 442 were valid, effective rate is 96.09%. The Pittsburgh sleep quality index (PSQI) score of 442 clinical nurses was 7.07 ± 2.14, of which 60 (13.57%) had sleep disorders; the Generalized Anxiety Disorder Scale (GAD-7) score was 4.77 ± 3.50, of which 182 (41.18%) had varying degrees of anxiety; The score of PHQ-9 was 5.95 ± 3.79, of which 187 (42.31%) had different degrees of depressive symptoms. The stepwise multiple linear regression analysis which involved PHQ-9 and GAD-7 scores showed that: both the PHQ-9 score and the GAD-7 score were positively correlated with the sleep quality score, and the PHQ-9 score increased every time 1 point, sleep quality score increased by 0.239 points; GAD-7 score increased by 1 point, sleep quality score increased by 0.150 points. The overall model test (*F* = 109.760, *P* < 0.001) regression model is meaningful.

**Conclusion:**

Decreased sleep quality is common among clinical nurses in infectious disease hospitals, and the sleep status of nurses is positively correlated with anxiety and depression. Nursing managers pay attention to sleep quality of clinical nurses in infectious disease hospitals and carry out effective interventions to improve the sleep quality of nurses.

## Introduction

Infectious disease specialist hospitals are responsible for the diagnosis, treatment, and prevention of infectious diseases. Due to the infectious and epidemic characteristics of infectious diseases, the risk of infection faced by their front-line nurses is much higher than that of other types of hospitals, so that nurses in infectious disease specialist hospitals face greater pressure than other groups (Wang and Dong, 2013; Zhou, 2016). Since December 2019, due to the worldwide pandemic of the new coronavirus, doctors and nurses have been facing great work pressure, especially medical staff in the infectious disease department, who need to quickly adapt to intense critical care situations and a large number of critically ill patients, a large number of patient deaths, and the risk of infection (Marvaldi et al., 2021).

It is well known that the quality of care is closely related to the mental state of health workers. Studies have shown that Chinese nursing staff have different degrees of sleep problems (Cai et al., 2021; Cheng et al., 2021; Ding et al., 2021; Guo et al., 2021; Yin et al., 2021). Lack of sleep will not only cause physical discomfort but also increase the psychological burden of nurses, thus adversely affecting nursing work (Xiao et al., 2021). At present, research on sleep quality of nurses mainly focuses on personal and family factors, workload, shifts, etc. (Li et al., 2021; Liu et al., 2021). There are few studies on the relationship between sleep status and anxiety and depression of clinical nurses in infectious disease specialist hospitals. Therefore, to understand the current status of sleep quality of nurses in infectious disease hospitals and analyze its influencing factors, so as to reduce the psychological pressure of nurses in infectious disease specialist hospitals, improve their sleep conditions, provide better services for patients, and promote the development of infectious disease prevention and treatment. This study investigated the sleep status of clinical nurses in a large tertiary infectious disease specialist hospital and analyzed its influencing factors, in order to provide some reference and basis for improving their sleep quality.

## Objects and methods

### Research objects

This retrospective study was conducted by using the convenience sampling method. 460 clinical nurses from a tertiary infectious disease specialist hospital were selected as the research objects in September 2021. Inclusion criteria: (1) Obtained and registered as a nurse professional qualification; (2) currently working in the clinical front line; (3) working time ≥ 3 years; (4) consent and voluntary participation in our study. Exclusion criteria: (1) training personnel; (2) vacation personnel; (3) history of psychiatric or neurological disorders that could interfere with participation in the study.

### Research tools

General information was obtained on the age, sex, marital status, years of experience, types of institution (hospital or primary setting), and comorbidities of participants *via* a sociodemographic questionnaire (Al Maqbali, 2021).

#### Pittsburgh sleep quality index

PQSI was compiled in 1989 by Dr. Buysse, a psychiatrist at the University of Pittsburgh, United States. This scale is widely used to assess sleep quality. The scale has 19 items and 7 dimensions, which are subjective sleep quality, sleep latency, sleep duration, habitual sleep efficiency, sleep disturbances, use of sleep medications, and daytime dysfunction. The score for each of the seven components can range from 0 to 3. The PSQI global score is calculated by summing the seven components, which a possible range from 0 to 21, with a global score of ≥ 6 indicating poor sleep quality in the previous month (Dong et al., 2020; Al Maqbali, 2021). The higher the score, the worse the subject’s sleep quality. In this study, the scale has good reliability (0.80) and validity (0.864) in a previous study of Chinese population (Lu et al., 2014), and is widely used in the assessment of sleep quality in various populations (Fabbri et al., 2021; Moriana et al., 2022).

#### The Generalized Anxiety Disorder Scale

GAD-7 was compiled, and was recommended to the whole country by the Psychiatry Branch of the Chinese Medical Association on June 8, 2015. There are 7 items in GAD-7, and the score is the sum of all items: 0–4 points for anxiety without clinical significance; 5–9 points for mild; 10–14 points for moderate; > 15 points for severe (Xiang et al., 2018; Byrd-Bredbenner et al., 2021; Lee et al., 2021; Zinchuk et al., 2021). The scale is a self-rating scale with simple content, strong operability, which had been verified in previous studies with good reliability (0.90) and validity (0.94) (AlSwailem et al., 2021), and is widely used in anxiety assessment of various populations.

#### The Depression Screening Scale

Patient Health Questionnaire-9 (PHQ-9) is 9 items based on the diagnostic criteria of DSM-IV (Diagnostic and Statistical Manual of Mental Disorders developed by the American Psychiatric Association). The subjects’ feelings in the past 2 weeks were evaluated on a scale of 0 (not at all) to 3 (almost every day), with a total score ranging from 0 to 27. The higher the score, the more severe the depressive symptoms. This is a simple and effective self-rating scale for depression, which has good reliability (0.955) and validity (0.979) in Chinese patients in terms of auxiliary diagnosis of depression and assessment of symptom severity (Shen Y. et al., 2021).

### Survey methods

In this study, the questionnaire survey method was used, and the researchers used the questionnaire star software^1^ to distribute and collect the questionnaires online. These self-administered questionnaire were revised by 5 epidemiologists and occupational health experts and piloted in 32 emergency nurses. Before issuing the questionnaires, a notice of filling in the questionnaire was issued to each department to obtain their informed consent, and the subjects were introduced to the research purpose, significance, and matters needing attention when filling in the questionnaire using a unified guide language. All items are set as mandatory questions, and the questionnaire filling method is set to limit one answer per device to avoid repeated answers.

### Statistical methods

SPSS 25.00 software was used for statistical analysis in this study; quantitative variables were tested for normality (Shapiro-Wilk test), and measurement data subject to normal distribution were described by mean ± standard deviation (X ± S), and single-sample was used for statistical description. The *t*-test was used to compare the differences between the quantitative indicators and the norm; the two independent samples *t*-test and analysis of variance were used to compare the groups; the Pearson correlation analysis was used to compare the correlation between the scores; the multiple linear regression model was used to analyze the influencing factors of sleep quality; For all analyses, the level of significance was set at *P* < 0.05.

## Results

### General information

A total of 442 valid questionnaires were recovered, with an effective recovery rate of 96.09%. Among the 442 clinical nurses, 15 were male (3.39%), and 427 (96.61%) were female; 148 (33.4%) aged 18–25, 150 (33.9%) aged 26–55, and 145 aged 56 and above (32.8%); 111 college students (25.11%), 329 undergraduate students (74.43%), and 2 master students (0.45%).

### Sleep quality, anxiety, and depression of clinical nurses in infectious disease specialist hospitals

Among the 442 clinical nurses, 382 had sleep quality less than or equal to 7 points, 60 had sleep quality greater than 6 points, and 13.57% of the nurses had sleep problems; In GAD-7, 260 (58.82%) with 0–4 points, 134 (30.31%) with 5–9 points, 29 (6.5%) with 10–14 points, 19 (4.3%) with 15–21 points, of which 182 nurses (41.18%) had different degrees of anxiety symptoms; In PHQ-9, 255 (57.69%) with 0–4 points, 125 (28.28%) with 5–9 points, 38 (8.6%) with 10–14 points, and 16 (3.6%) with 15–19 points, of which 187 (42.31%) nurses had different degrees of depressive symptoms (Table 1).

**TABLE 1:** Survey results of sleep quality and anxiety and depression among nurses in infectious disease specialist hospitals.

Scale	Degree	Frequency	Percentage	−X ± S
PSQI	Good sleep quality (<6 points)	382	86.43	7.07 ± 2.14
	Poor sleep quality (≥6 points)	60	13.57	
GAD-7	Normal (0–4 points)	260	58.82	4.77 ± 3.50
	Mild (5–9 points)	134	30.32	
	Moderate (10–14 points)	29	6.6	
	Severe (15–21 points)	19	4.3	
PHQ-9	Normal (0–4 points)	255	57.69	5.95 ± 3.79
	Mild depression (5–9 points)	125	28.28	
	Moderate depression (10–14 points)	38	8.6	
	Moderate to severe depression (15–19 points)	16	3.6	
	severe depression (20–27 points)	8	1.8	

PSQI, Pittsburgh sleep quality index; GAD-7T, Generalized Anxiety Disorder-7; PHQ-9, Patient Health Questionnaire-9.

### Comparison of scores of sleep quality, depression, and anxiety under different conditions

The differences in the scores and total scores of each factor of sleep quality, PHQ-9 and GAD-7 total scores in gender, age and education were compared. The results showed that there was no significant difference between the genders in the rest of the score indicators except that the males took longer to fall asleep than the females (*P* < 0.05). There was no significant difference in sleep disturbance and daytime function among different ages (*P* > 0.05). The rest of the score indicators basically showed a slightly higher score in the middle age group (*P* < 0.05). In the comparison of scores among different educational backgrounds, the scores of the high educational group were higher than those of the low educational group (*P* < 0.05) (Table 2).

**TABLE 2:** Comparison of sleep quality, depression and anxiety.

Index	Sleep quality	Time to fall asleep	Sleeping time	Sleep disorder	Hypnotize drug	Daytime features	PSQI	PHQ-9	GAD-7
**Sex**									
Male	1.02 ± 0.69	1.13 ± 0.76	1.16 ± 0.78	0.90 ± 0.52	0.02 ± 0.15	1.75 ± 0.78	6.92 ± 2.00	5.73 ± 3.69	4.56 ± 3.27
Female	1.05 ± 0.69	0.96 ± 0.76	1.27 ± 0.91	0.95 ± 0.57	0.03 ± 0.18	1.88 ± 0.84	7.23 ± 2.27	6.18 ± 3.88	4.99 ± 3.72
*T*	–0.427	2.303	–1.309	–1.098	–0.632	–1.640	–1.522	–1.252	–1.298
*p*-value	0.672	0.022	0.192	0.273	0.528	0.102	0.129	0.211	0.195
**Age**									
18–25	0.97 ± 0.69	0.96 ± 0.74	1.15 ± 0.91	0.85 ± 0.58	0.01 ± 0.12	1.80 ± 0.82	6.73 ± 1.78	5.32 ± 3.31	4.43 ± 3.23
26–55	1.17 ± 0.75	1.18 ± 0.85	1.39 ± 0.89	0.97 ± 0.53	0.06 ± 0.24	1.93 ± 0.85	7.93 ± 2.67	7.32 ± 4.10	6.08 ± 3.87
≥56	0.97 ± 0.62	1.00 ± 0.68	1.10 ± 0.69	0.95 ± 0.52	0.01 ± 0.08	1.71 ± 0.75	6.56 ± 1.53	5.19 ± 3.56	3.78 ± 2.93
*F*	4.121	3.606	5.124	2.125	4.838	2.666	19.435	15.589	18.288
*p*-value	0.017	0.028	0.006	0.121	0.008	0.071	<0.001	<0.001	<0.001
**Education**									
College and below	0.92 ± 0.72	0.90 ± 0.74	1.06 ± 0.72	0.86 ± 0.55	0.01 ± 0.09	1.64 ± 0.77	6.18 ± 1.68	5.23 ± 3.52	4.32 ± 3.07
Undergraduate	1.13 ± 0.68	1.06 ± 0.74	1.41 ± 0.99	0.90 ± 0.53	0.05 ± 0.21	1.87 ± 0.86	7.45 ± 2.21	6.63 ± 3.74	5.07 ± 3.79
Postgraduate	1.05 ± 0.67	1.17 ± 0.79	1.16 ± 0.76	1.01 ± 0.55	0.03 ± 0.16	1.91 ± 0.77	7.51 ± 2.21	5.93 ± 3.97	4.88 ± 3.56
*F*	3.466	4.671	6.667	2.858	2.115	4.545	18.777	5.028	1.752
*p*-value	0.032	0.010	0.001	0.058	0.122	0.011	<0.001	0.007	0.175

PSQI, Pittsburgh sleep quality index; GAD-7, Generalized Anxiety Disorder-7; PHQ-9, Patient Health Questionnaire-9; T, True; F, False.

### Comparison of sleep quality, anxiety, and depression scores among people with and without sleep problems

The total score of PSQI < 6 points was regarded as the group without sleep problems (*n* = 273), and the total score of PSQI ≥ 6 points was regarded as the group with sleep problems (*n* = 169). The PSQI factor scores, anxiety and depression scores of the patients were higher than those of the no sleep problem group, and the difference was statistically significant (*P* < 0.05) (Table 3).

**TABLE 3:** Comparison of scores of sleep quality, depression, and anxiety among people with and without sleep problems.

Index	No sleep problems (*n* = 273, %)	With sleep problems (*n* = 169, %)	χ^2^/t	*p*-value
Sleep quality	0.86 ± 0.66	1.33 ± 0.64	–7.309	<0.001
Time to fall asleep	0.81 ± 0.70	1.43 ± 0.70	–8.920	<0.001
Sleeping time	0.92 ± 0.69	1.69 ± 0.85	–9.974	<0.001
Sleep disorder	0.82 ± 0.53	1.10 ± 0.52	–5.502	<0.001
Hypnotic drugs	0.00 ± 0.00	0.07 ± 0.26	–3.583	<0.001
Daytime features	1.60 ± 0.71	2.15 ± 0.85	–7.387	<0.001
Total PSQI socre	5.77 ± 1.16	9.19 ± 1.60	–24.095	<0.001
Total PHQ-9 score	4.03 ± 2.36	9.06 ± 3.60	–16.160	<0.001
Total GAD-7 score	3.41 ± 2.39	6.97 ± 3.88	–10.737	<0.001

PSQI, Pittsburgh sleep quality index; GAD-7, Generalized Anxiety Disorder-7; PHQ-9, Patient Health Questionnaire-9.

### Correlation analysis

Comparing the 7-dimension score and total score of PSQI, as well as the difference between PHQ-9 score and GAD-7 score, the results show that there is a basic positive correlation between GAD-7 score, with PSQI and PHQ-9 score, respectively (Table 4).

**TABLE 4:** Correlation analysis of sleep quality, depression and anxiety scores.

Index	Sleep quality	Time to fall asleep	Sleeping time	Sleep disorder	Hypnotic drugs	daytime Features	PSQI	PHQ-9	GAD-7
Sleep quality	1	0.169**	0.203**	0.025	0.172**	0.174**	0.408**	0.294**	0.261**
Time to fall asleep	0.169**	1	0.202**	0.211**	0.172**	0.066	0.457**	0.287**	0.276**
Sleeping time	0.203**	0.202**	1	0.094*	0.221**	0.290**	0.488**	0.352**	0.284**
Sleep disorder	0.025	0.211**	0.094*	1	0.177**	–0.001	0.254**	0.153**	0.130**
Hypnotic drugs	0.172**	0.172**	0.221**	0.177**	1	0.142**	0.326**	0.223**	0.178**
Daytime features	0.174**	0.066	0.290**	–0.001	0.142**	1	0.462**	0.294**	0.269**
Total PSQI socre	0.408**	0.457**	0.488**	0.254**	0.326**	0.462**	1	0.534**	0.438**
Total PHQ-9 score	0.294**	0.287**	0.352**	0.153**	0.223**	0.294**	0.534**	1	0.455**
Total GAD-7score	0.261**	0.276**	0.284**	0.130**	0.178**	0.269**	0.438**	0.455**	1

PSQI, Pittsburgh sleep quality index; GAD-7, Generalized Anxiety Disorder-7; PHQ-9, Patient Health Questionnaire-9. *P < 0.05; **P < 0.01.

### Multiple linear regression model analysis of factors affecting sleep quality of nurses in infectious disease specialist hospitals

The PHQ-9 and GAD-7 scores were included in the multiple linear regression model, and the factors influencing the total score of sleep quality were analyzed according to input = 0.05 and output = 0.10, and a stepwise multiple linear regression analysis was performed. The results showed that: PHQ-9 score and GAD-7 score were both positively correlated with sleep quality score, and the sleep quality score increased by 0.239 points for every 1 point increase in PHQ-9 score. Every time the GAD-7 score increased by 1 point, the sleep quality score increased by 0.150 points. Model overall test regression model is meaningful (*F* = 109.760, *P* < 0.001) (Table 5).

**TABLE 5:** Fitting results of multiple linear regression models.

Variables	Partial regression coefficient	Standard error	Standardized Partial regression coefficients	T	*p*-value	95% CI
Constants	4.941	0.167			0.000	(4.613, 5.268)
PHQ-9	0.239	0.025	0.423	9.666	0.000	(0.190, 0.287)
GAD-7	0.150	0.027	0.245	5.603	0.000	(0.097, 0.202)

GAD-7, Generalized Anxiety Disorder-7; PHQ-9, Patient Health Questionnaire-9.

## Discussion

As the health care workers who have the most frequent contact with patients, nurses have a very heavy workload. In China, due to the large number of patients and the shortage of medical staff resources, the workload of nurses is particularly heavy (Shen Y. et al., 2021). Several domestic and foreign studies have shown that the mental state of health care workers during the COVID-19 pandemic is characterized by high levels of anxiety (23.2–26%) and depression (25–22.8%) (Krishnamoorthy et al., 2020; Luo et al., 2020; Pappa et al., 2020; Wu et al., 2020). Similar to the results of this study, the external validity of this study was further improved. Analysis of related factors showed that: PHQ-9 score and GAD-7 score were both positively correlated with sleep quality score, and the sleep quality score increased by 0.239 points for every 1 point increase in PHQ-9 score. Every time the GAD-7 score increased by 1 point, the sleep quality score increased by 0.150 points (Table 5). The possibility of the presence of probable insomnia in nurses with reduced sleep quality. Therefore, improving the anxiety and depression symptoms of nurses in infectious disease hospitals can improve their sleep quality.

The results of this study showed that the PSQI index of clinical nurses in infectious disease specialist hospitals was 7.07 ± 2.14, which was a relatively high level in existing relevant studies (Shen L. F. et al., 2021; Zhou et al., 2021); male nurses took longer to fall asleep than females; Except for daytime function, the other indicators all showed the characteristics of slightly higher scores in the middle age group; the scores of the high-education group were higher than those of the low-education group (Table 2). The sleep status of nurses was positively correlated with anxiety and depression, that is, the more severe the anxiety and depression, the more obvious decline in sleep quality (Table 3).

It may be caused by the following reasons. First of all, the nature of work in infectious disease hospitals is special, and nurses have been exposed to high-risk environments for a long time, resulting in greater psychological pressure (Serrão et al., 2022). Nurses were identified as one of the professional groups experiencing the highest levels of stress (Lee and Kim, 2020). Improper coping with pressure will affect the sleep quality of nurses, reduce the quality of nursing, and lead to job burnout. Salilih and Abajobir (2014) studied 343 nurses, 37.8% of whom reported that they experienced stress related to work shifts, illness and other factors. Nursing requires intense concentration and independent thinking to make health-related decisions for the patient. Anxiety affects the ability of nurses to make appropriate decisions (Polat et al., 2019), which leads to errors and decreased quality of care. Second, infectious disease specialist hospitals are not understood in society due to the particularity of their admissions, and due to the public’s fear of infectious diseases, nurses in infectious disease specialist hospitals lack a sufficient sense of social identity (Ruiz-Fernández et al., 2020); Third, the number of male nurses in clinical work is relatively small, and there is a lack of same-sex communication and professional identity; Finally, in addition to the clinical work, the highly educated group also undertakes more non-clinical work, and the work pressure is relatively high.

There are two levels of stress solution: macro level and micro level (Maunder et al., 2008). The macro level refers to government and hospital initiatives, while the micro level refers to measures taken by individuals and individuals to relieve stress. At the macro level (i.e., official organizational measures), the work environment in terms of hospital safety and satisfaction is an important factor affecting perceived stress (Goh et al., 2015). It is essential to ensure that protective materials are used adequately and effectively to create a conductive working environment. At the same time, adequate professional protection training is also an urgently needed intervention. Accordingly, interventions might include securing their leisure time or providing cognitive behavioral therapy (Shen Y. et al., 2021). For nurses working in isolation wards, in the absence of offline training, we suggest that nursing managers pay attention to the psychological response of nurses and use the online platform to hold protective training and psychological assistance courses.

The main limitation of this study is the use of convenience sampling. This sampling method limits the sample size and health care population, and may introduce sampling bias that affects the potential generalizability of the findings. In addition, since there are far more female nurses in hospitals than male, this created gender differences in the participants (males 3.39% vs. females 96.61%) that could not be artificially avoided. Another limitation is the lack of testing data on the same population before the COVID-19 outbreak, which makes it difficult to determine the direct impact of the outbreak on the mental health of nurses in infectious disease specialist hospitals. Future research should focus on using the same measurement tools to compare and contrast symptoms of depression, anxiety, and stress among nurses in infectious disease specialist hospitals during and after the COVID-19 pandemic.

## Summary

To sum up, decline in sleep quality is common among clinical nurses in infectious disease specialist hospitals, especially since the epidemic of COVID-19 in 2019. It is recommended to strengthen the occupational protection level of nurses in infectious disease specialist hospitals, conduct regular physical examinations, and monitor the physical health of nurses. Regularly conduct mental health education lectures, set up mental health consultation rooms, form a long-term mechanism for mental health surveys and psychological crisis intervention of nursing staff, and guide clinical nurses to take positive ways to deal with work pressure. Strengthen humanistic care, create a relaxed and pleasant working atmosphere, affirm the contributions and efforts of nurses in infectious disease specialist hospitals, and allow them to obtain sufficient social support and professional identity.

## Data availability statement

The original contributions presented in this study are included in the article/supplementary material, further inquiries can be directed to the corresponding author/s.

## Ethics statement

The studies involving human participants were reviewed and approved by the Beijing Youan Hospital of China Capital Medical University. The patients/participants provided their written informed consent to participate in this study.

## Author contributions

SX and YG conceived of the study. HG and BJ participated in its design and coordination. FG, WM, and LZ helped to draft the manuscript. All authors read and approved the final manuscript.

## Footnotes

1
https://www.wjx.cn/
